# A Comprehensive Evaluation of miR-144-3p Expression and Its Targets in Laryngeal Squamous Cell Carcinoma

**DOI:** 10.1155/2021/6684186

**Published:** 2021-07-16

**Authors:** Bin-Yu Mo, Jin-Shu Pang, Wen-Bin Dai, Ya-si Su, Wei Jiang, Su-Ning Huang

**Affiliations:** ^1^Department of Otolaryngology, Liuzhou People's Hospital of Guangxi, No. 8 Wenchang Rd., Liuzhou, Guangxi Zhuang Autonomous Region, 545006, China; ^2^Department of Radiotherapy, Guangxi Medical University Cancer Hospital, No. 71 Hedi Rd., Nanning, Guangxi Zhuang Autonomous Region, 530021, China; ^3^Department of Pathology, Liuzhou People's Hospital of Guangxi, No. 8 Wenchang Rd., Liuzhou, Guangxi Zhuang Autonomous Region, 545006, China; ^4^Department of Educational Administration, Guangxi Medical University Cancer Hospital, No. 71 Hedi Rd., Nanning, Guangxi Zhuang Autonomous Region, 530021, China

## Abstract

Laryngeal squamous cell carcinoma (LSCC) is an aggressive type of head and neck squamous cell carcinoma (HNSCC) with a relatively high rate of morbidity and mortality. An altered miR-144-3p level in LSCC with a small number of patients has been previously reported. However, the clinical implication of miR-144-3p and its involved mechanism underlying this disease is not clearly elucidated. In this work, we aimed to confirm the expression of miR-144-3p with larger samples and also to identify target genes for the investigation of the underlying mechanism of miR-144-3p in LSCC. The levels of miR-144-3p were downregulated in 155 samples of LSCC tissues as compared to 26 non-LSCC samples (SMD: -0.78; 95% confidence interval (CI): -1.23, -0.32). The AUC of 0.90 in the summarized ROC curve also indicated a potential ability to differentiate LSCC from non-LSCC tissues, with a sensitivity of 0.78 and a specificity of 0.88. With respect to the molecular mechanism, we predicted the potential targets from online-based prediction, peer-reviewed publications, and RNA-seq and microarray data. In particular, the genes influenced by transfection with miR-144-3p in the LSCC FaDu cell line were collected from the microarray GSE56243. Lastly, 12 novel targets for miR-144-3p in LSCC were obtained by different algorithms. In conclusion, our study confirmed the loss or downregulation of miR-144-3p in LSCC, which might contribute to the LSCC tumorigenesis and progression via regulation of the 12 novel targets, such as IL24, ITGA6, and CEP55. In the future, further investigations are required to validate the present results.

## 1. Introduction

Laryngeal squamous cell carcinoma (LSCC) is the most common type (>95%) of laryngeal cancer and has a relatively high rate of morbidity and mortality [1]. Multiple factors, such as excessive alcohol and smoking, have been associated with the aetiology of carcinogenesis and the progression of LSCC [2]. In the past decades, although researchers have made some progress regarding the early diagnosis and therapeutic strategy of LSCC, numerous LSCC patients still face unfavourable clinical outcomes [2]. Therefore, further investigation on the mechanism of LSCC remains crucial.

MicroRNAs are crucial endogenous regulatory factors that usually exert a potent influence in various pathological processes of tumorigenesis and progression of human tumors by targeting sequence-specific genes [3]. Several microRNAs, such as miR-196b and miR-17-5p, have been considered markers for the early diagnosis and prognosis of LSCC patients [4–6]. Furthermore, miR-144-3p has also recently gained much attention for its distant alteration in various human cancers [7–9]. Chen et al. confirmed a marked loss or downregulation of miR-144-3p in lung malignancy [10]. Additionally, another study also demonstrated a lower miR-144-3p level in cervical cancer samples compared to a normal sample [11]. As for LSCC, downregulated miR-144-3p has been reported as performing a tumor-suppressive role by targeting insulin receptor substrate 1 (IRS1) and E26 transformation specific-1 (ETS1) [12, 13]. However, only a small sample size was tested to show the expression level of miR-144-3p in LSCC, and the molecular mechanism involved in miR-144-3p remains unknown due to the fact that altered miRNA influences a series of downstream genes amid the evolution of malignancy. Thus, this paper is aimed at confirming the expression of miR-144-3p in LSCC with 155 samples of LSCC tissues and 25 samples of non-LSCC mucosa and at further identifying additional target genes to investigate the underlying mechanism of miR-144-3p in LSCC.

## 2. Materials and Methods

### 2.1. Screening of Microarrays for miR-144-3p Expression and Its Targets

To investigate the miR-144-3p levels in LSCC and its potential targets, we searched for available data in public databases (Sequence Read Archive (SRA), ArrayExpress, and Gene Expression Omnibus (GEO)) as follows: (laryngeal OR pharyngeal OR “head AND neck” OR LSCC OR HNSCC) AND (RNA OR miRNA OR microRNA) AND (tumor OR cancer OR carcinoma OR neoplas∗ OR malignan∗). The features of the microarrays were as follows: (1) the datasets were based on human LCSS and normal mucosa tissues, and (2) there were more than three cases of tissues in both the LSCC and non-LSCC groups. [14]. In addition to microarrays, the RNA-sequencing data from the TCGA project were also utilized to evaluate the differences of miR-144-3p expression between LSCC and non-LSCC mucosa.

### 2.2. Statistical Analysis

All expression data were possessed with a normalization of log2(*x* + 1) scale. Student's *t*-test was used to measure the differences between two groups in GraphPad Prism 8, and one-way ANOVA analysis was performed for the differences among three or more groups. In Stata 12.0, a random effects model was calculated to estimate the overall expression of miR-144-3p when heterogeneity > 50%; otherwise, the fixed effects model was used. Moreover, in combination with a summary receiver operating characteristic (SROC) curve, the sensitivity and specificity forest plots as well as positive and negative diagnostic likelihood ratio (DLR) were used to measure the potential of miR-144-3p in diagnosing LSCC. The results were recognized as statistically significant when *p* < 0.05. [15]

### 2.3. Acquisition of Targets of miR-144-3p

To determine the putative targets of miR-144-3p, a computerized-based prediction was conducted using miRWalk 2.0, which included 12 software programs: miRDB, TargetScan, miRWalk, Miranda, miRBridge, PicTar, RNA22, Microt4, miRNAMap, PITA, RNAhybrid, and miRMap. We selected the genes that overlapped in three prediction software programs. To analyze the genes directly influenced by miR-144-3p in LSCC, a double-channel GSE56243 (GSM1357599) microarray was used, and it identified the differentially expressed genes (DEGs) after overexpressing miR-144-3p in LSCC Fadu cell lines. From the GSE56243 microarray, the genes with a log2 fold change (FC) < −1 were selected as candidate targets of miR-144-3p for subsequent analysis. Moreover, we further excavated DEGs between LSCC and non-LSCC controls from microarrays and TCGA RNA-seq data using the limma package and edgeR of the R language program, respectively. The genes that were differentially expressed in at least three datasets were recorded. Finally, the DEGs appearing for the aforementioned prediction methods were selected as the putative target genes of miR-144-3p in LSCC [16].

### 2.4. Molecular Mechanism for the Targets of miR-144-3p

After the acquisition of targets for miR-144-3p, gene ontology (GO) annotation in combination with pathway analysis based on the KEGG and Reactome pathway databases was performed to investigate the mechanism underlying LSCC [17]. The protein levels for these genes in LSCC were further verified by immunohistochemistry (IHC) according to the Human Protein Atlas (HPA). Considering few studies for IL24 in LSCC, in-house IHC experiments were performed to validate IL24 protein levels using 30 cases of LSCC tissues and 15 non-LSCC squamous epithelium. This research related to human tissues had been approved by the Ethics Committee of the Liuzhou People's Hospital of Guangxi (Liuzhou, China). The results of IHC staining were assessed by the percentage of positive cells and staining intensity as previously described [18]. And the protein levels of IL24 between LSCC and non-LSCC tissues were calculated Student's *t*-test.

## 3. Results

### 3.1. miR-144-3p Was Lowly Expressed in the LSCC

**Figure 1:**
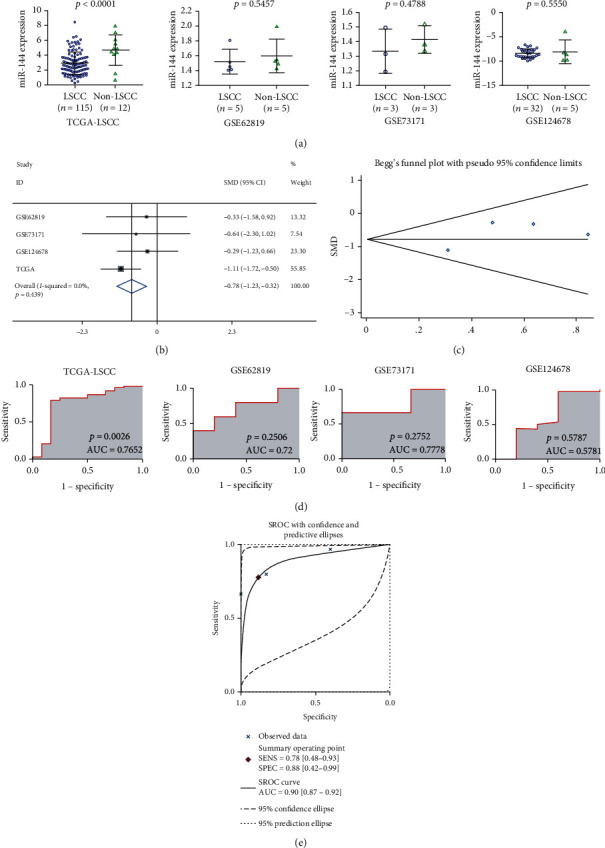
The expression of miR-144-3p and its clinical significance in LSCC and non-LSCC samples. (a) The expression differences of miR-144-3p in each study; (b) the pooled level of miR-144-3p in LSCC evaluated by random effects model; (c) Begg's test for the publication bias of included studies; (d) ROC curves indicating the potential ability of miR-144-3p in distinguishing LSCC from non-LSCC samples based on each study; (e) and summarized ROC curve indicating the potential ability of miR-144-3p in distinguishing LSCC from non-LSCC samples by integrating four studies.

A total of four datasets were eligible for this study, and they are comprised of 155 samples of LSCC tissues and 26 samples of non-LSCC mucosa (Fig. S1, Table 1). As depicted in Figure 1(a), the downregulation or loss of miR-144-3p in LSCC was clearly observed in comparison with non-LSCC tissues from RNA-seq data. Furthermore, consistent results were obtained from the fixed effects model, indicating that miR-144-3p exhibited a lower expression in the LSCC tissues, with an SMD of -0.78 (95% CI: -1.23, -0.32; *I*^2^ = 0.0%, Figure 1(b)). Moreover, no significant publication bias was detected by Begg's plot, which also strengthened the accuracy of the fixed effects model (*p* > 0.05, Figure 1(c)).

### 3.2. Clinical Value of miR-144-3p Levels in LSCC

**Figure 2:**
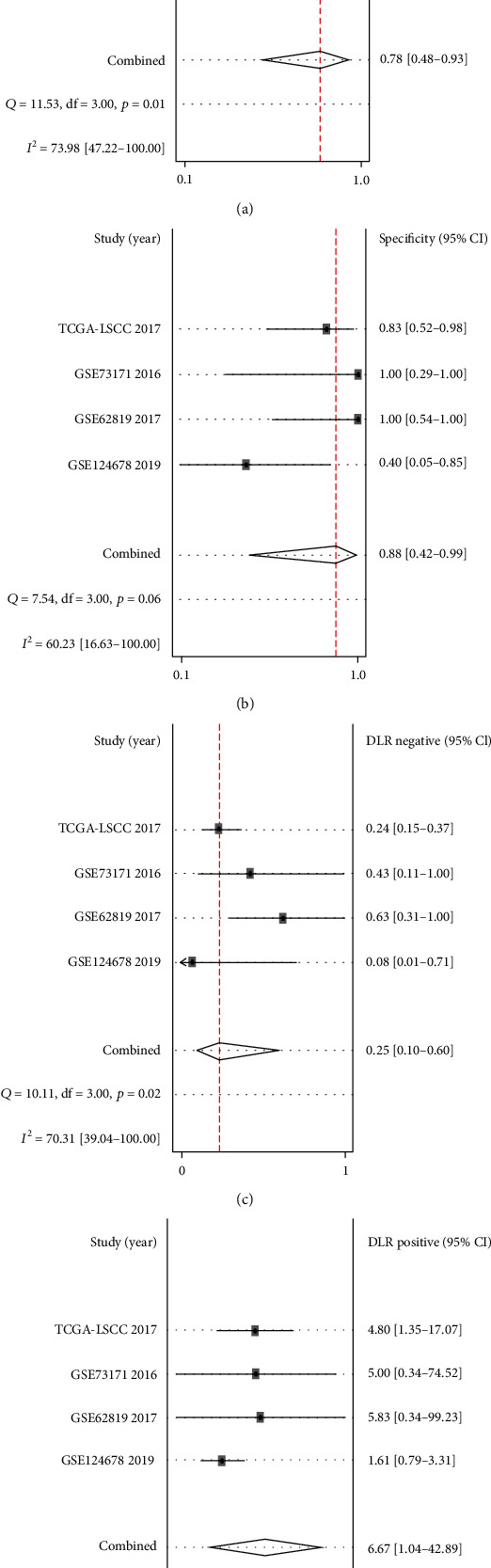
The clinical significance of miR-144-3p in LSCC. Sensitivity (a) and specificity (b) values of each included study. DLR negative (c) and DLR positive (d) of each included study.

**Figure 3:**
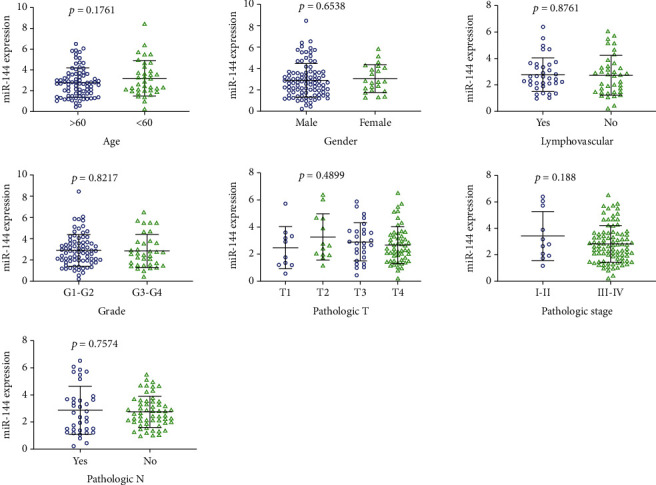
The expression difference between miR-144-3p levels and clinical features in LSCC.

In terms of diagnostic value, results suggested that miR-144-3p was a useful indicator for distinguishing LSCC from non-LSCC mucosa with an AUC of 0.9 detected by the SROC curve (Figures 1(d) and 1(e)). The results of sensitivity, specificity, negative DLR, and positive DLR were 0.78, 0.88, 0.25, and 6.67, respectively, which also indicated that miR-144-3p might act as a means of diagnosing LSCC (Figure 2). However, statistical analysis did not demonstrate any significant difference between miR-144-3p expression and clinical features in LSCC (Figure 3).

### 3.3. Targets of miR-144-3p in LSCC

**Figure 4:**
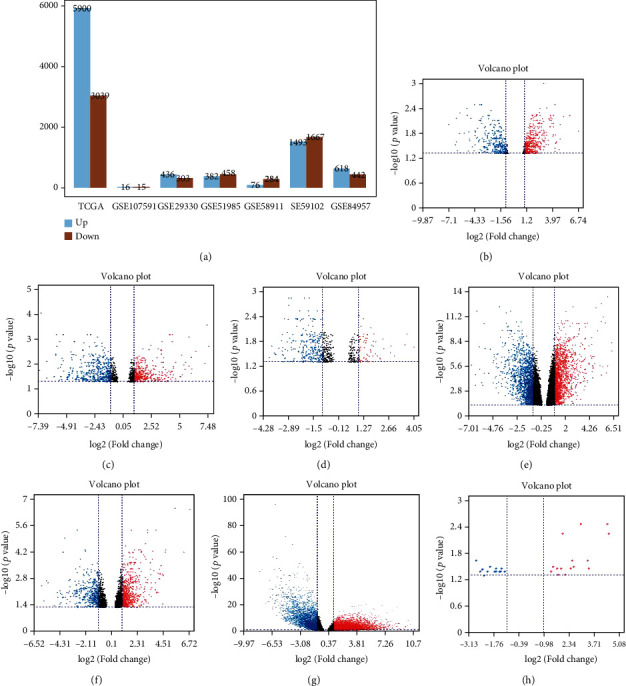
The differently expressed genes (DEGs) between LSCC and non-LSCC samples. (a) The overview of DEGs in LSCC; and volcano plots for GSE29330 (b), GSE51958 (c), GSE58911 (d), GSE59102 (e), GSE84957 (f), TCGA (g), and GSE10591.

**Figure 5:**
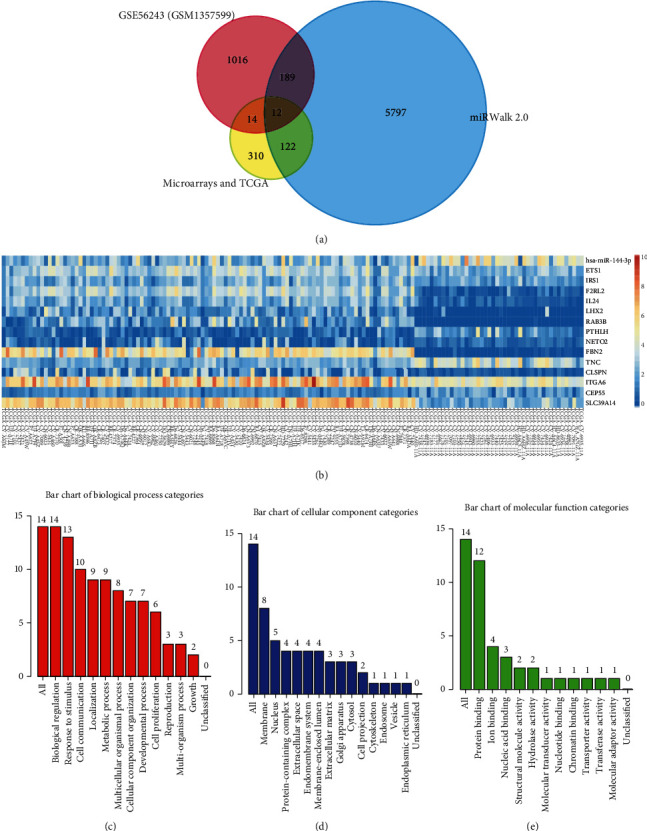
The identification for the targets of miR-144-3p and Gene Ontology (GO) analysis. (a) Microarrays and TCGA upregulating DEGs appearing for at least three datasets; miRWalk 2.0, genes appearing for at least three prediction software; GSE56143 (GSM1357599) recorded the information of genetic changes after transfection of miR-144-3p in LSCC cells, which was used for more accurate identification of targets of miR-144-3p; (b) heat map for the 14 targets of miR-144-3p in LSCC and non-LSCC samples; biological process (c), cellular component (d), and molecular function (e) of GO analysis.

From the integrative DEGs of the seven datasets (Figure 4, Table 2), 458 upregulated genes overlapping in three datasets were considered to be the candidate targets of miR-144-3p in LSCC (Figure 5). In addition, there were 6,120 genes potentially influenced by miR-144-3p from the online-based prediction by miRWalk 2.0. Furthermore, 1,231 genes were significantly downregulated when miR-144-3p were transfected into LSCC Fadu cells. Eventually, 12 genes appearing at TCGA and Affymetrix datasets, online predicting software, and microarray after miR-144 transfection were regarded as the most likely prospective targets of miR-144 (Figure 5(b)). In addition, ETS1 and IRS1 were also included for this study as a result of previous reports [12, 13].

### 3.4. Gene Ontology and Pathway Analysis

For the biological process of GO annotation (Figures 5(c)–5(e)), targets of miR-144-3p are associated with response to stimulus, cell communication, and metabolic processes. According to cellular components, the proteins coded by these genes were located in the membrane, nucleus, and protein-containing complex. Regarding molecular function, targets of miR-144-3p appeared to play roles in protein binding, iron binding, and nucleic-acid binding. With respect to KEGG and Reactome pathway analysis (Table 3), ITGA6 and TNC were noticeably involved in multiple pathways, such as syndecan interactions, nonintegrin membrane-ECM interactions, and PI3K-Akt signalling pathway.

### 3.5. Further Investigation for the Targets of miR-144-3p

**Figure 6:**
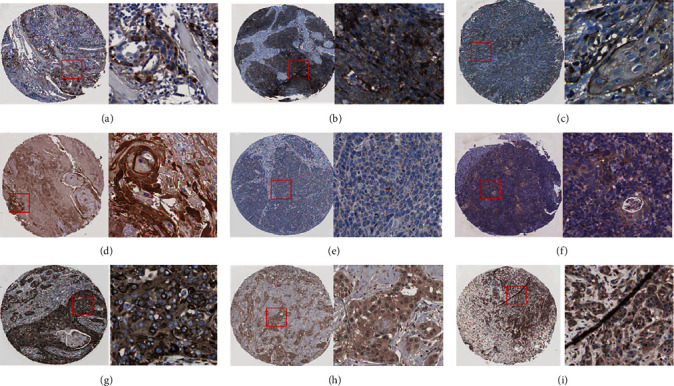
The protein levels for the targets of miR-144-3p in LSCC provided by the Human Protein Atlas. (a) TNC protein detection using HPA004823 antibody; (b) ITGA6 protein detection using HPA012696; (c) NETO2 protein detection using HPA013180; (d) CEP55 protein detection using HPA023430; (e) RAB3B protein detection using CAB023293; (f) PTHLH protein detection using HPA035982; (g) SLC39A14 protein detection using HPA016508; (h) IL24 protein detection using CAB025972; (i) IRS1 protein detection using CAB005261. The protein levels of FBN2, F2RL2, ETS1, LHX2, and CLSPN were not detected by the Human Protein Atlas. Note: protein levels of non-LSCC tissues were not detected, and these results were just used to show the potential protein levels of targets of miR-144-3p in LSCC rather than the expression difference.

**Figure 7:**
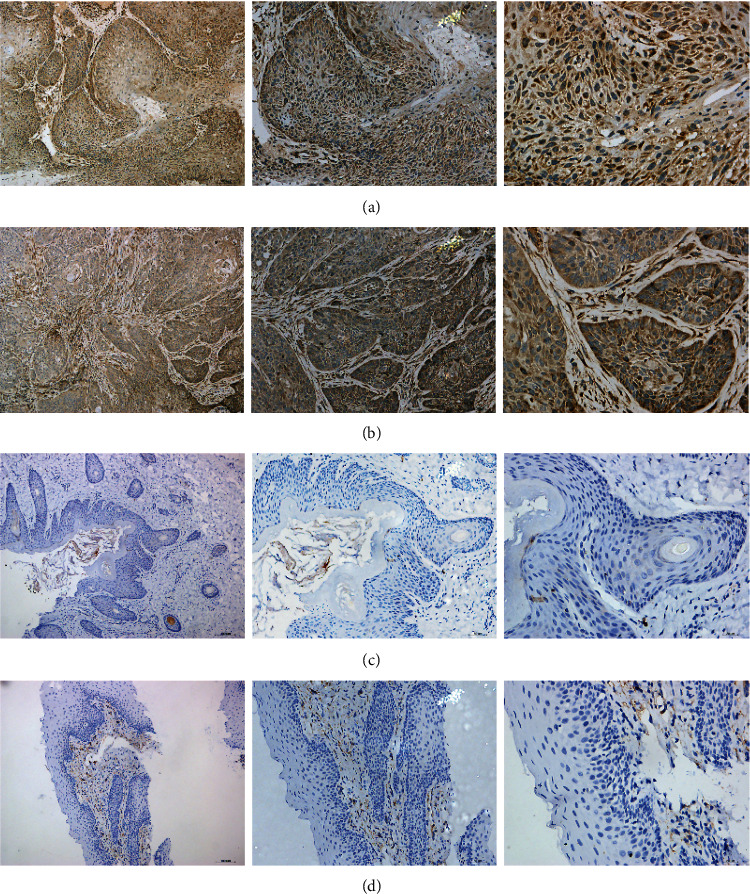
The in-house IHC staining results for IL24 protein in LSCC (a; ×100, ×200, and ×400), papilloma (b; ×100, ×200, and ×400), and non-LSCC epithelium (c; ×100, ×200, and ×400).

The mRNA expression of these 14 genes was upregulated in LSCC tissues compared with non-LSCC tissues, among which 9 protein expressions could be detected by IHC in LSCC in the HPA databases (Figure 6). Besides, in-house IHC were further performed to validate the expression of IL24 protein. According to the evaluation of IHC staining scores, IL24 protein levels were obliviously overexpressed in 30 cases of LSCC tissues compared with 15 non-LSCC squamous epithelium (Table 4). And IHC staining results for LSCC, papilloma, and non-LSCC epithelium are displayed in Figure 7.

## 4. Discussion

This study investigated the downregulation of miR-144-3p and its clinical significance in malignant LSCC using 155 samples of LSCC tissues and 26 samples of non-LSCC mucosa. Furthermore, the putative targets of miR-144-3p were also determined using multiple databases, followed by an in silico analysis, including pathway and GO enrichment analyses for the investigation of underlying mechanisms.

LSCC deriving from laryngeal epithelial cells is an aggressive type of head and neck carcinoma [1]. In several studies, miR-144-3p has been considered an accelerator that promotes tumor deterioration and has been found to be overexpressed in head and neck carcinoma [19, 20]. However, some studies have also suggested downregulated miR-144-3p in LSCC in comparison with non-LSCC mucosa. A study published in 2016 demonstrated that a lower miR-144-3p expression was exhibited in LSCC compared to non-LSCC mucosa and that low levels of miR-144-3p inhibited cell growth and distant migration of LSCC cells by directly targeting IRS1 [12]. In addition, downregulated miR-144-3p was reported as having the ability to suppress tumors by overexpressing ETS1 [13]. However, the miR-144-3p expression in these studies was only examined by RT-qPCR and used only a few samples. In this study, we confirmed the expression level of miR-144-3p in LSCC with larger samples by integrating miRNA-seq data and miRNA profiles. The pooled result demonstrated that miR-144-3p was clearly decreased in the 155 samples of LSCC tissues as compared with the 26 non-LSCC mucosa samples. As for the potential effectiveness of miR-144-3p to distinguish LSCC from non-LSCC mucosa, the present results revealed that downregulated miR-144-3p may eventually serve as a useful marker in the diagnosis of malignant LSCC with a summarized AUC of 0.9. Nevertheless, more experiments based on other factors, such as serum and saliva, are needed for confirmation of the diagnostic ability of miR-144-3p regarding LCSS. Furthermore, it was rather disappointing that no evidence was found supporting any relation between miR-144-3p and clinicopathological features in LSCC.

After verifying the expression of miR-144-3p in LSCC, in silico analysis was used to elaborate the specific molecular mechanism involved. Previous studies have reported that downregulated miR-144-3p could influence several targets to accelerate carcinogenesis and cancer deterioration of LSCC [12, 13]. For example, increased miR-144-3p could clearly suppress IRS1 levels, thereby inhibiting PI3K/AKT pathway activation. However, additional targets of miR-144-3p in LSCC are worth exploring. To improve the reliability of targets, we identified the targets of miR-144-3p by integrating five elements, including peer-reviewed publication, online-based prediction, miR-144-3p transfection microarray, TCGA miRNA-seq data, and GEO microarray profiles. Notably, transfection microarray recorded the gene alteration in LSCC cells after overexpressing miR-144-3p. In total, 14 genes were finally regarded as the most likely targets of miR-144-3p in LSCC. Of these 14 genes, two (viz., ITGA6 and TNC) contributing to several pathways are worth paying attention to. Interestingly, the findings provided by Zhu's study revealed that ITGA6 may be directly targeted by miR-144-3p in cervical cancer and that lncRNA ATB promoted the viability of cell proliferation and invasion through the miR-144/ITGA6 axis [21]. Furthermore, the protein levels of TNC in the stroma have been proven to be an excellent prognostic marker in oral cancer patients, suggesting that five-year survival rate was 88% when stromal TNC was negative; however, for cases with overexpression of TNC, it decreased to 43% [22]. Furthermore, it should be mentioned that our published study revealed that IL24 protein was highly expressed in LSCC tissues and exerted an influence in LSCC tumorigenesis [18]. Furthermore, some findings based on pathway analysis suggested that targets of miR-144-3p may be active in driving LSCC carcinogenesis and deterioration through syndecan interactions and nonintegrin membrane-ECM interactions pathways. As previously reported, syndecan serves as (co)receptors to influence cell signalling and cell behavior, which play roles in angiogenesis, tissue regeneration, lipid metabolism, and even pathogenesis of several diseases [23–25]. Syndecans are also involved in human cancers [26]. Increased syndecan-1 in lung microenvironments accelerated the outgrowth of mammary carcinoma metastases, whereas higher syndecan-1 levels inhibited lung carcinogenesis by regulating exosome miRNAs, and lung cancer patients with higher syndecan-1 levels were more likely have better prognoses [27, 28]. Furthermore, nuclear translocation of syndecan-1 has been also reported to mediate TGF-*β* pathway activation and several transcription factors, thereby suppressing the growth of fibrosarcoma cells [29]. However, no research has yet thoroughly investigated the correlations between syndecan interactions and LSCC. The results of this study may provide novel insights into drug discovery and clinical decision-making regarding LSCC.

Although the results performed were generally successful, the study included several limitations. Specifically, the targets of miR-144-3p in this study were obtained only from in silico methods. Regarding the targets, the focus of our study is to provide a discovery of another 12 novel targets using different algorithms rather than pathways that miR-144-3p might regulate, which need to be carefully considered with in vitro experiments. In addition, although big sample data based on evidence-based methods were applied to explore the expression of miR-144-3p, experiments should be performed to investigate miR-144-3p's molecular mechanism. Moreover, the diagnostic effectiveness and clinical utility of miR-144-3p regarding LSCC require further exploration.

In short, this study provides more evidence for the downregulation of miR-144-3p in LSCC and suggests that downregulated miR-144-3p may serve as a useful diagnostic indicator to differentiate LSCC from non-LSCC tissues. Moreover, the loss or downregulation of miR-144-3p contributes to LSCC tumorigenesis and progression via regulation of the 12 novel targets, such as IL24, ITGA6, and CEP55. Further future investigation is required to validate and expand on the present results.

## Figures and Tables

**Table 1:** Details of the included studies for miR-144-3p expression in LSCC.

Study	Year	HNSCC/non-HNSCC	Cancer types	Exp. mean ± SD	Con. mean ± SD
GSE62819	2017	5/5	LSCC	1.524 ± 0.170	1.587 ± 0.210
GSE73171	2016	3/3	LSCC	1.334 ± 0.152	1.415 ± 0.094
GSE124678	2019	32/5	LSCC	−8.427 ± 0.9115	−8.086 ± 2.432
TCGA	2017	115/12	LSCC	2.913 ± 1.522	4.662 ± 2.045

**Table 2:** Details of studies for identification of DEGs between LSCC and non-LSCC tissues.

Accession	Platform	Subtype	Cancer numbers	Normal numbers
TCGA	/	LSCC	115	12
GSE29330	GPL570	LSCC	13	5
GSE51985	GPL10558	LSCC	10	10
GSE59102	GPL6480	LSCC	29	13
GSE84957	GPL17843	LSCC	9	9
GSE58911	GPL6244	LSCC	15	15
GSE107591	GPL6244	LSCC	24	23

**Table 3:** The pathway analysis using the 14 targets of miR-144-3p in LSCC.

Gene set	Description	Size	*p* value	Genes
R-HSA-3000170	Syndecan interactions	2	0.00041	ITGA6, TNC
R-HSA-3000171	Nonintegrin membrane-ECM interactions	2	0.001957	ITGA6, TNC
hsa04512	ECM-receptor interaction	2	0.003367	ITGA6, TNC
R-HSA-216083	Integrin cell surface interactions	2	0.004174	ITGA6, TNC
R-HSA-1474244	Extracellular matrix organization	3	0.004174	ITGA6, TNC, FBN2
hsa04151	PI3K-Akt signalling pathway	3	0.005336	ITGA6, TNC, IRS1
R-HSA-74713	IRS activation	1	0.005674	IRS1
hsa05206	MicroRNAs in cancer	2	0.01091	IRS1, TNC
hsa04510	Focal adhesion	2	0.01872	ITGA6, TNC
hsa04960	Aldosterone-regulated sodium reabsorption	1	0.040011	IRS1

**Table 4:** IL24 protein levels between LSCC and non-LSCC tissues.

Terms	Tissues	*n*	Mean ± SD	*t*	*p* value
Statistical analysis	LSCC	30	8.733 ± 1.701	15.11	<0.001
	Non-LSCC	15	1.333 ± 1.175		

## Data Availability

The data used to support the findings of this study are included within the article.

## Supplementary Materials

Supplementary Materials Fig. S1: the flow chart for the selection of the included studies for miR-144-3p expression (A) and the identification of DEGs between LSCC and non-LSCC (B). Fig. S2: the expression levels for the 14 targets of miR-144-3p using RNA-seq data.
