# Supplementary material for: A Comprehensive Evaluation of miR-144-3p Expression and Its Targets in Laryngeal Squamous Cell Carcinoma
Source: Comput Math Methods Med. 2021 Jul 16;2021:6684186. doi: 10.1155/2021/6684186 (PMC8302387; doi:10.1155/2021/6684186)
Supplement: Supplementary Materials — Fig. S1: the flow chart for the selection of the included studies for miR-144-3p expression (A) and the identification of DEGs between LSCC and non-LSCC (B). Fig. S2: the expression levels for the 14 targets of miR-144-3p using RNA-seq data. [file 6684186.f1.doc]

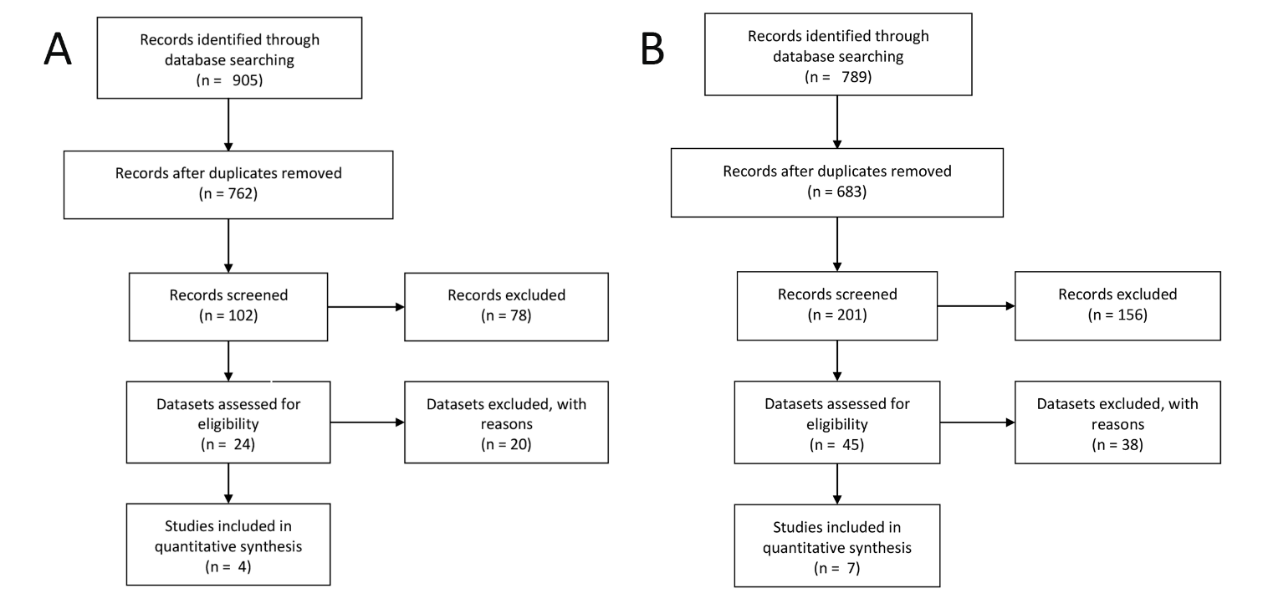


Fig. S1 The flow chart for the selection of the included studies for miR-144-3p expression (A) and the identification of DEGs between LSCC and non-LSCC(B).


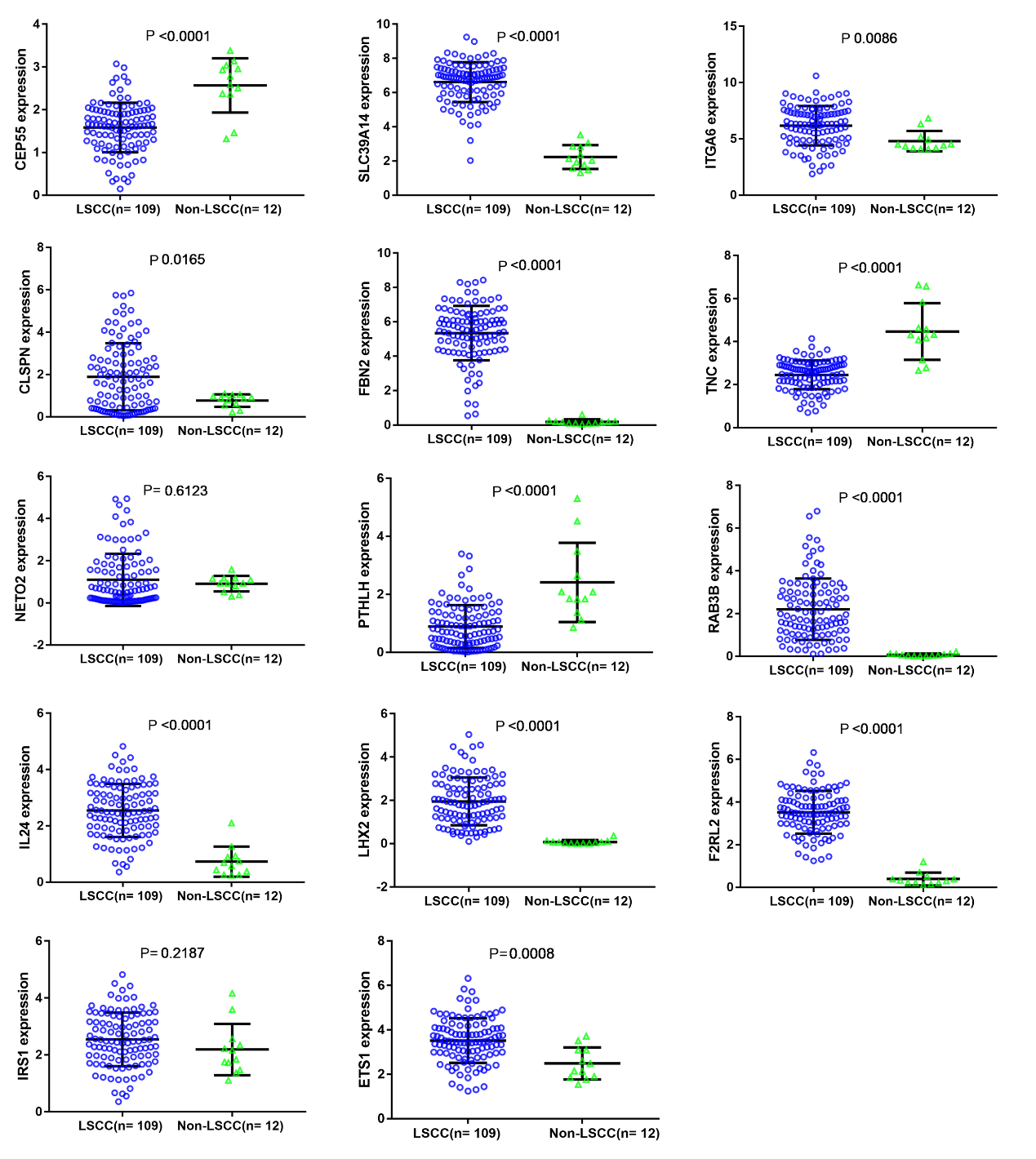


Fig. S2 The expression levels for the 14 targets of miR-144-3p using RNA-seq data.
